# Importance for bilateral palpation of pulse old fact rediscovered

**DOI:** 10.4103/0019-5049.63636

**Published:** 2010

**Authors:** Namita Saraswat, Amrita Gupta, Abhijeet Mishra, Uma Srivastava

**Affiliations:** Department of Anaesthesiology, PG Student, S N Medical College, Agra, India; 1 Professsor, S N Medical College, Agra, India

Sir,

A 20-year-old female patient, weighing 30 kg, of ASA status I, was scheduled for laparoscopic cholecystectomy under general anaesthesia. On conducting a preanaesthetic check up two days prior, no abnormality was detected and laboratory investigations were within normal limits. In the operation theatre an IV line was established on the right forearm and monitors were applied. When the pulsoximetry probe was put on left index finger it did not pick up the signals. Non-invasive blood pressure also could not be measured on the left arm. Thereafter all peripheral pulses including radial, brachial, axillary and subclavian were checked and they were not palpable on the left side, whereas, peripheral pulses on the right side were palpable, but were of low volume. Both carotids and lower limb pulses were normal. The non-invasive blood pressure measured in the right arm was 100 / 70 mm Hg and pulse rate was 86 / minute. The electrocardiogram (ECG) was normal. The patient was again enquired, but no abnormal symptoms (such as, dizziness, vertigo, visual changes, transient ischaemic attack (TIA), or stroke)[1] could be detected. The differential diagnosis of diminished or absent pulses in one limb were thought to be due to cervical rib, peripheral vascular diseases, vasculitis, such as, Takayasu arteritis, or diseases like atherosclerosis. However, according to the age and signs we presumed the probable cause as Takayasu arteritis. The probable diagnosis and risk of surgery were explained to patient and attendants. But as insisted by the patient and attendants we decided to proceed with the surgery keeping in mind the problems associated with Takayasu arteritis (avoidance of hyperextension of the cervical spine and maintaining perfusion pressure of the vital organs).[2,3] Standard general anaesthesia was induced with sodium pentothal, midazolam and fentanyl. Endotracheal intubation was facilitated with vecuronim bromide. Anaesthesia was maintained with O_2_ in 60% N_2_O, halothane and vecuronium. The surgery lasted for 45 minutes and the intraoperative course was uneventful. We investigated the patient postoperatively. X-ray of the chest and ECG were normal. The Colour Doppler showed diffuse wall thickening of the proximal and middle parts of left and right subclavian arteries with severe luminal narrowing on the left side and partial luminal narrowing on the right side. Damped (reduced) blood flow was seen in the distal arterial system of the left upper limb. Thickening in the wall of the abdominal aorta was also seen.

We missed the diagnosis of Takayasu arteritis, because radial pulses were not palpated on both sides. We are reporting this case just to make the anaesthetist more vigilant and re-emphasize the importance of the old fact of bilateral palpation of pulses during physical examination.
